# Aphid populations and virus vector potential in potato fields across seasons and regions in Norway

**DOI:** 10.1038/s41598-025-20355-5

**Published:** 2025-10-21

**Authors:** Nina Svae Johansen, Hans Geir Eiken, Simeon Lim Rossmann, May Bente Brurberg, Monica Skogen, Marta Bosque Fajardo, Borghild Glorvigen, Toril Sagen Eklo, Finn-Arne Haugen, Snorre Hagen, Erik Lysøe

**Affiliations:** 1 Division of Biotechnology and Plant Health, Norwegian Institute of Bioeconomy Research, 1431 Ås, Norway; 2 Division of Environment and Natural Resources, Norwegian Institute of Bioeconomy Research, Ås, Norway; 3 Faculty of Biosciences, Norwegian University of Life Sciences, Ås, Norway; 4 Division of Survey and Statistics, Norwegian Institute of Bioeconomy Research, Ås, Norway; 5 Norwegian Agricultural Extension Service (NLR), Gjesåsen, Norway

**Keywords:** Aphididae, Barcoding, COI, Phylogenetics, Virus vectors, Agroecology, Biodiversity, Microbial ecology, Population dynamics

## Abstract

Several aphid species pose serious treats to potato crops by causing direct damage to the plants and/or indirectly by transmitting viruses. Different morphological forms and phenotypic plasticity among aphids complicates taxonomy and identification and thus makes targeted pest management in potatoes challenging. To obtain an overview of aphids frequenting potato fields in Norway, we investigated seasonal and annual changes in aphid populations in five potato fields (58–64 °N) over a three-year period (2016–2018), using yellow pan traps. In total 2218 of the 6136 collected aphids were identified by traditional barcoding, meaning sequencing a ~ 650 fragment of the mitochondrial *COI* gene. This revealed 137 different species, of which 111 were identified at the species level. The remaining were identified only to the genus level, indicating potential novel species. The southernmost sampling location yielded the highest number of species and individual counts, although no clear correlations to climate factors (temperature/precipitation) was observed. Of the 111 species identified, at least 39 are potential vectors of potato virus Y (PVY) and nine species may also transmit potato virus A (PVA). Knowledge on virus vector and non-vector aphid abundance and phenology have the potential to improve pest management of potato cultivation.

**Supplementary Information:**

The online version contains supplementary material available at 10.1038/s41598-025-20355-5.

## Introduction

Potato (*Solanum tuberosum*) is highly vulnerable to a wide range of pests and pathogens due to its tender foliage, underground tubers, and susceptibility to diseases. Different aphid species are among the pests that feast on potato. Aphids (Hemiptera: Aphididae) with over 5200 species in more than 500 genera are in general economically important insect pests of many agricultural and forest crops^1,2^. These small, soft-bodied insects feed on most plant parts, with approximately 250 species impacting agricultural and horticultural crops^3^. Aphids inflict damage through direct feeding on plants and serve as vectors for various plant pathogens. They can also become highly invasive when carried by the wind in large swarms^4^. As a result, aphids are serious pests in temperate agricultural regions, such as Norway^5^.

Norway has recorded 329 aphid species through morphological identification, primarily from plants but also some from traps^6–8^. As a group, they are distributed throughout Norway, including all the major potato districts. More species are recorded in South-Norway than in the north^6–15^. Notably, nineteen aphid species and sub-species from the Palaearctic Region have potatoes as host plants^16^. Of these, eight species (*Aphis fabae, A. frangulae, A. nasturtii, Aulacorthum solani, Brachycaudus helichrysi, Macrosiphum euphorbiae, M. ascalonicus* and *M. persicae*) have been collected from potato fields in Norway^11–14,17^, and seven species (*Aphis crassivora, A. gossypii, A. rumicis, Jaksonia pappillata, Myzus ornatus, Rhopalosiphum nymphaeae* and *Rhopalosiphum padi*) from other host plants or traps in Norway. Recently, additional 27 species were morphologically identified amongst aphids collected in yellow pan traps in a three-year survey in potato in fields in South Norway^17^.

Over 40 aphid species including the notorious *Myzus persicae* and *Macrosiphum euphorbiae* are known virus vectors in potato fields^18–21^. The aphid-transmitted viruses potato virus Y (PVY), potato virus A (PVA), and potato leaf roll virus (PLRV) cause major economic losses in potato and seed potato production worldwide^22–24^. PVY poses the greatest threat in most countries, while PVA significantly impacts Northern Europe, North-Central USA, and Canada^24^. Norway strictly regulates the maximum allowable incidence of PVY and PVA in certified seed potatoes. PVY and PVA are transmitted in a non-persistent, stylet-borne manner by various aphid species^22,25^. The most damaging potato virus, PLRV, is persistently transmitted, but was eradicated from Norway in the 1950’s^22^.

Controlling aphids is an important strategy for virus control in potato. Monitoring the flight activity of both potato-colonizing and non-colonizing key aphid species is used to predict risk of PVY and PVA-transmission, guiding timing of aphid treatment and haulm killing in potato crops^26^. The risk assessment often relies on species-specific relative efficiency factors, estimated from the aphid species’ capability to transmit PVY, *Myzus persicae* is considered the most efficient vector and is thus used as a reference species^27–29^. Instantaneous virus pressure (at a specific time) and cumulative vector pressure are estimated from the abundance and diversity of the key species throughout the cropping season. Several studies suggest that the total number of aphids that land and probe on the potato crop during its peak susceptibility period, rather than number of certain key species, may better explain PVY infection and spreading^22,24^. A better understanding of aphid diversity and seasonal phenology is essential for developing an effective potato virus risk warning system, thus quick and reliable aphid identification is essential. Identifying aphid species by morphology alone is complicated, time consuming, and often impossible, due to their complex life cycles, with different morphological forms influenced by alternating plant host selection, environmental factors, and high species diversity^30,31^. The DNA sequence of the 5’region of the mitochondrial *cytochrome c oxidase 1* (*COI*) gene has proven to be highly effective for aphid species identification^4,32^. The sequencing process is referred to as DNA barcoding and has become the preferred method for characterising biodiversity^33^. Its usefulness and accuracy in biodiversity studies rely on several important steps such as the number of samples, the geographical spread of samples, the size and quality of the sequence database^32^. For Aphididae, the number of *COI* sequences in BOLD is ~ 100.000 sequences (September 2024). The utility of DNA barcodes in aphid species determination have recently been evaluated, and Li et al.^32^ found that a threshold value of ≥ 98% genetic distance is suitable for distinguishing most species. Genetic diversity within species may be found for some species because of genetic divergence through differing selection pressures such as alternating host plants or geographical isolation^32^. In addition, genomic studies have also documented introgression events between aphid species that disturb the phylogenetic relationships^31^.

In this study, we have used DNA barcoding to identify aphid species in five potato fields in Southern Norway, and to investigate changes within and between years over a 3-year sampling period, relative to location and weather conditions. We aimed to investigate seasonal, annual, and spatial trends, to increase the knowledge of the aphids that immigrate to potato fields in Norway as a base for improving management of PVY and PVA.

## Methods

### Aphid sampling

**Fig. 1:**
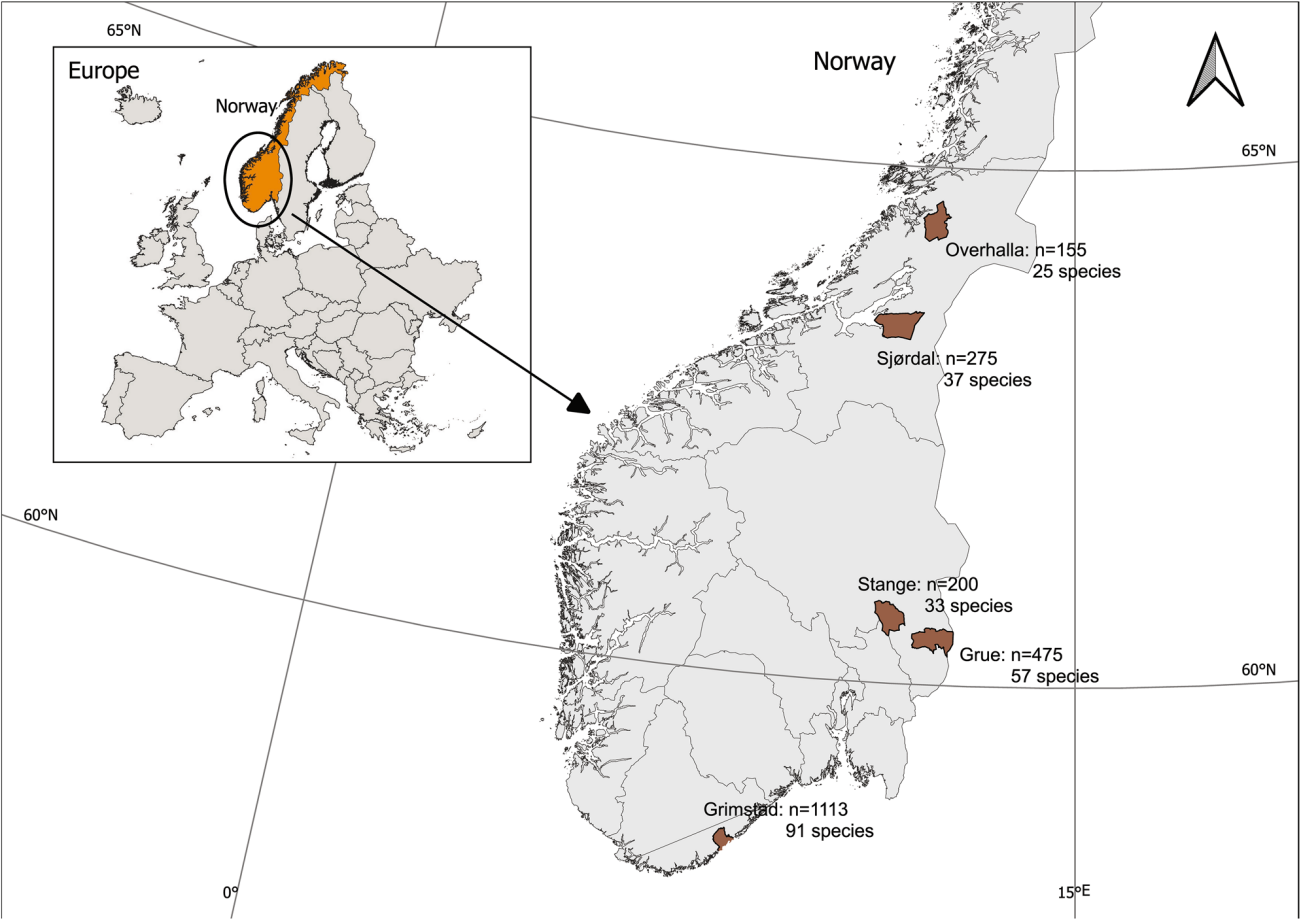
Numbers of aphids barcoded (n) and number of species identified from the five different potato fields in Norway. Aphids were sampled in the growing seasons 2016–2018 in Grimstad (11–15 sampling weeks), Grue (10–14 sampling weeks) and Stjørdal (11–12 sampling weeks), and in 2016–2017 in Stange (10–12 sampling weeks) and Overhalla (12–13 sampling weeks). The number of sampling weeks corresponded to the potato growing period at the different sites and years. The maps were created with QGIS 3.34. 11-Prizren using basemap Norway © Norwegian Mapping Authority / Geonorge (administration unit municipalities). Source: https://kartkatalog.geonorge.no/metadata/administrative-enheter-kommuner/041f1e6e-bdbc-4091-b48f-8a5990f3cc5b, and basemap Europe © Norwegian Mapping Authority/Geonorge (Europa illustration map). Source: https://kartkatalog.geonorge.no/metadata/europa-illustrasjonskart/825eab8c-9f97-4a21-8424-d51f0b02cada.

**Fig. 2:**
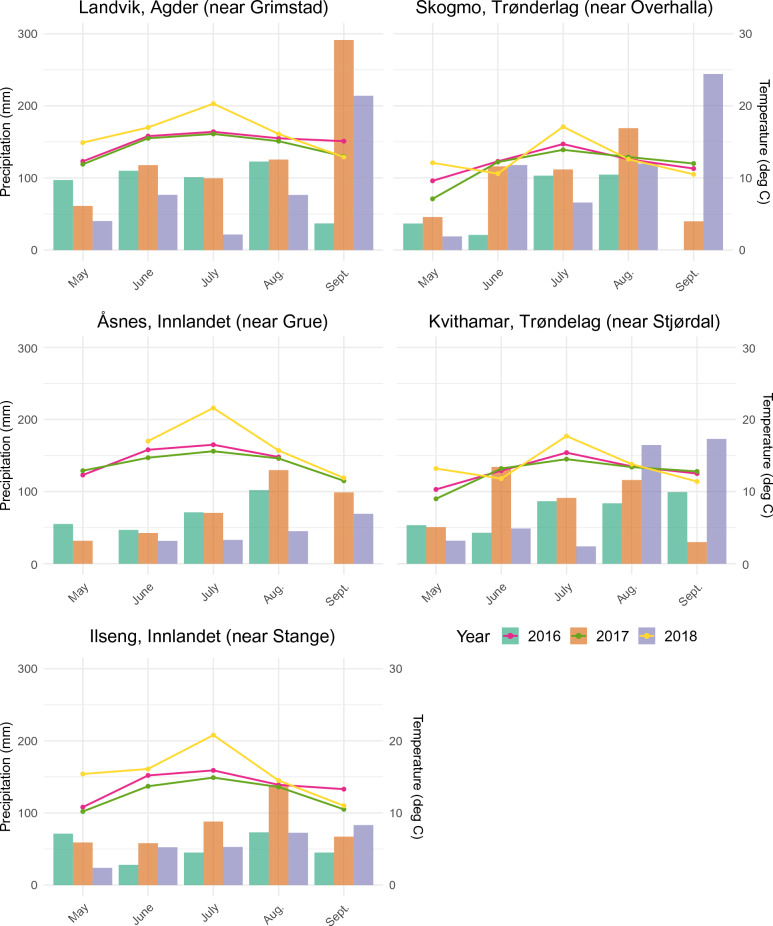
Monthly mean air temperature 2 m above ground level and total monthly precipitation during the growing seasons 2016–2018 at the nearest weather station to the collection sites Landvik (Agder), Åsnes (near Innlandet), Ilseng (Innlandet), Skogmo (Trøndelag) and Kvithamar (Trøndelag). Data from Agrometeorology Norway-LMT.

Samples were collected from five potato fields in South-, Southeast-, and Mid-Norway, ranging from 58 to 64 °N: Grimstad (Agder), Grue and Stange (Innlandet) and Stjørdal and Overhalla (Trøndelag) in the period 2016–2018 (Fig. 1). The landscape at all locations is structured by agricultural areas bordered by mixed forests of varying ages. Temperature and precipitation sums obtained from Agrometeorology Norway, in the periods from potato seedling emergence to haulm killing are shown in Table 1. Monthly mean air temperature (measured at 2 m height) and precipitation totals for each location and year are shown in Fig. 2.

**Table 1:** **N**umber of aphids collected and barcoded from potato fields at different locations in Norway in the period 2016–2018, together with geographic and weather data.

Site and year	Temperature sum T_o_ = 0 °C	Precipitation sum (mm)	Trapping period	Nearest weather station altitude	Total number of aphids	Number of barcoded aphids
Grimstad 2016	1200	302	03/06–17/08	Landvik 10 m	2716	1113
Grimstad 2017	1654	362	22/05–05/09			
Grimstad 2018	1560	170	04/06–30/08			
Grue 2016	1220	213	20/06–05/09	Åsnes 155 m	1145	475
Grue 2017	1155	212	16/06–30/08			
Grue 2018	1798	160	08/06–19/09			
Stange 2016	1236	137	24/06–14/09	Ilseng 182 m	1017	200
Stange 2017	1041	229	15/06–25/08			
Stjørdal 2016	1024	184	22/06–30/08	Kvithamar 28 m	864	275
Stjørdal 2017	1101	314	12/06–30/08			
Stjørdal 2018	1232	223	04/06–27/08			
Overhalla 2016	1127	220	10/06–31/08	Skogmo 32 m	394	155
Overhalla 2017	1210	401	07/06–06/09			

The sum of temperature measured 2 m above ground and precipitation are from the nearest weather stations to the collection sites during the aphid flight monitoring periods.

Aphid flight activity was monitored with yellow pan traps (length 43 cm, width 28 cm, height 11 cm), designed specifically for trapping alates in their landing mode^34^. Each field/site contained one trap, centrally placed in a 6.4 × 18.0 m (115.2 m^2^) plot with eight rows of potato varieties ‘Asterix’ (2016–2018), ‘Mandel’ (2016–2018), ‘Innovator (2016) or ‘Lady Claire’ (2017–2018). The plots were separated from the surrounding potato field by a 1.5 m boundary of bare soil. Each trap was filled with 5–7 L tap water with 1–2 tablespoons of dishwashing detergent (Zalo, Orkla, Ski, Norway) to break water surface tension, and 25 g of sodium benzoate and benzoic acid (Tørsleffs Atamonpulver, Haugen-gruppen AS, Vestby, Norway) to prevent fungal and bacterial growth.

Monitoring started in May–June (week 21–27, depending on year and site) approximately one week before the potato plants emerged. The traps were then emptied every week until haulm destruction in August–September (week 33–37). The volume of water in the traps was adjusted according to precipitation and evaporation. The trap height was raised so that the traps’ upper surface was approximately in line with the top of the plant canopy, ensuring exposure to immigrating aphids throughout the growing season. Watering, fertilization, and control of late blight of potato and weeds in the experimental fields were done following local agricultural practices. Insecticides were not used.

Aphids were collected from the pan traps by first pouring the content of water and insects through a fine-meshed sieve, and then transfer the insects left in the sieve into a 0.5 L bottle with tap water and one tablespoon Tørsleffs Atamonpulver for transport to the laboratory. Here, the aphids were extracted from the samples, sorted under a binocular microscope (115 × magnifying) based on their morphological traits^2,3,9–14^, transferred to sample tubes containing 70% alcohol and then stored at 3 °C in darkness awaiting barcoding. A total of 2544 of the 6136 collected individuals (37.2% of the total number of aphids caught in the traps) were selected for DNA barcoding after the following procedure: In sample tubes that contained ≤ five specimens, all aphids were barcoded. In sample tubes with > five specimens, five aphids were randomly extracted and barcoded.

### DNA Extraction, PCR, and barcoding

DNA was extracted from the aphids as described by^35^. For barcoding, the universal primers LCO1490/HCO2198 (5'-GGTCAACAAATCATAAAGATATTGG-3'/5'-TAAACTTCAGGGTGACCAAAAAATCA-3')^36^ were used to amplify a 658-bp fragment of the *COI* gene. PCR amplification was carried out with 3 µl of the DNA extract as template in a 25 µl PCR reaction on a Biorad, T100 Thermal Cycler. PCR conditions for amplification were as follows: initial denaturation for 1 min at of 95 °C, followed by 5 cycles of [94 °C for 40 s, 45 °C for 40 s, 72 °C for 1 min], then 35 cycles of [94 °C for 40 s, 51 °C for 40 s, 72 °C for 1 min] and a final extension of 72 °C for 7 min. The PCR reagents consisted of 1 × PCR buffer (Invitrogen), 1.5 mM of MgCl_2_ (Invitrogen), 0.2 mM of the dNTP mixture (Thermo Scientific), 0.2 µM of each primer (Thermo Scientific) and 0.5 units of Platinum Taq polymerase (Invitrogen), per reaction. PCR-products were visualized and confirmed as single bands on a 1% agarose gel (Invitrogen) and sequenced in both directions by Eurofins Genomics in Germany. The sequences were trimmed and forward and reverse sequences were assembled using CLC Main Workbench 21 before using blastn to search the NCBI nucleotide (nt) database. Taxonomy was derived from the TaxID of the best hit with the NCBI taxdump (11.07.2022). MAFFT v7.453 was used to adjust sequence direction (adjust direction), and to create an alignment of all sequences (default settings)^37^. For single representative sequences of aphid species, raxmlHPC-PTHREADS-SSE3 version 8.2.12^38^ was used to make a maximum likelihood alignment with rapid bootstrap (-n 1000). FastTree v2.1.11 was used to make the phylogenetic trees that was visualized using iTOL v6^39,40^. Kimura-2 parameter model was used to calculate the intraspecific and interspecific genetic distance using MEGA 11^41^.

### Statistical analysis

We analyzed weekly aphid counts during 2016–2018 (N = 152 site-weeks, weeks 1–15) against temperature, precipitation, year, and site. Because repeated observations were taken from the same site in each year, the data are not independent. To avoid pseudoreplication and to account for this within-site correlation, we fitted a negative binomial generalized linear mixed model (GLMM) with a log link:

$${\text{Aphids}} \sim {\text{Temperature}}\, + \,{\text{Percipitation}}\, + \,{\text{factor}}\left( {{\text{Year}}} \right)\, + \,{\text{Location}}\, + \,(1\left| {{\text{Location}}:{\text{Year}}} \right.).$$

A random intercept for Location:Year captured within‑season correlation for repeated weeks in each site‑year stratum. Model fit was evaluated using AIC, BIC, log-likelihood, the NB2 dispersion parameter, and random-effect variance. Effect sizes are reported as log-scale coefficients (β) with SE, Wald z, and two-sided p-values. The statistical analysis was conducted in Excel^®^ 2016 and the software R version 4.3.2 (R Core Team, 2023) using the glmmTMB package. p values ≤ 0.05 were considered statistically significant.

## Results

### Cytochrome oxidase sequence analysis and phylogenetics

**Fig. 3:**
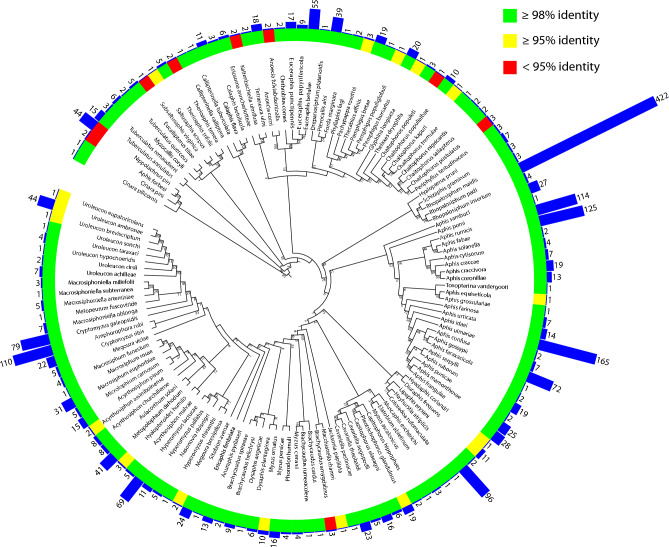
Phylogram of the best Maximum Likelihood tree determined by RaxML based on a ~ 650 fragment of the COI gene of 137 aphid species collected from potato fields in Norway. One single individual aphid was used to represent the species, and blue bars show number of aphids found in this study, in total 2218 specimens.

Out of a total of 6136 aphid specimens collected across five sites over three years, the *COI* gene was sequenced from 2218 (36.1%), resulting in the identification of 137 species. The datasets generated and analysed during the current study are available in the NCBI repository, accession numbers PV437596–PV439812. Of these, 111 were ≥ 98% identical to sequences in the NCBI nt database, which is the recommended threshold to distinguish between aphid species^32^ (Fig. 3, Table S1). The identified species belonged to nine sub-families (Anoeciinae, Aphidinae, Calaphidinae, Chaitophorinae, Drepanosiphinae, Eriosomatinae, Lachninae, Phyllaphidinae and Thelaxinae) and 54 genera. The remaining 26 species were identified to 21 genera within the sub-families Anoeciinae, Aphidinae, Calaphidinae, Chaitophorinae, Eriosomatinae, Lachninae and Thelaxinae). The sequence data showed a strong bias toward A + T content (G + C = 24.6%). Intraspecific genetic distances ranged from 0 to 7.5% with an average of 0.44% (Table S2). The largest intraspecific genetic distance appeared within *Eucallipterus tiliae* (five specimens). The interspecific genetic distance ranging from 0 to 19.53%, with an average distance of 6.2%. The largest interspecific genetic distances occurred between a specimen with closest match to the species *Anoecia fulviabdominalis* (blastn identity 92–93%) and *Chaitophorus saliapterus* (blastn identity 98.6%).

### Aphid abundance and species diversity

Between 2016 and 2018, a total of 6136 alate aphids were trapped at the five collection sites, spanning the period from potato seedling emergence in spring to haulm destruction in autumn. Aphid abundance was highest in the south and decreased northwards (Fig. 1; Table 1). Among the 2218 individuals (36.1%) identified with DNA barcoding, the highest abundance and species diversity were found in the southernmost location, and fewest in the northernmost collection site. The decline in abundance and diversity from south to north was correlated with sampling numbers. Based on the DNA barcoding results, *Aphis* was the genus with the highest number of species recorded, with 20, 13, 6, 10, and 6 species in Grimstad, Grue, Stange, Stjørdal, and Overhalla, respectively. *Aphis coronilla, A. fabae, A. gossypii, A. idaei,* and *A. rumicis* were found at all sites, and *A. fabae* was found in all years except in Overhalla in 2016. Certain species were site-specific: *Aphis confusa, A. cytisorum, A. grossulariae, A. punicae, A. ruborum, A. serpylli, A. taraxicola* and *A. ulmariae* were only recorded in Grimstad, *Aphis pomi* was only found in Grue, and *A. solanella* only in Stjørdal. Among the species identified at a blastn threshold of ≥ 98%, include 17 species that have earlier not been reported from Norway^6,7,9–15,17^. An overview of the species identified each year at the different sites is provided in Table S3.

Across years, the genus *Aphis* was most abundant in Grimstad (44.1%) followed by the species *Amphorophora rubi* (18.2%) and *Rhopalosiphum padi* (6.9%). In Grue, *Rhopalosiphum padi* was most abundant (28%) followed by the genus *Aphis* (21%), *Cavariella* (6.2%) and *Euceraphis* (6.0%). *Rhopalosiphum padi* was the most numerous species also in Stange (75%), Stjørdal (43%) and Overhalla (38%), followed by the genus *Aphis* which accounted for 5% of the total catch in Stange, 6% in Stjørdal and 7% in Overhalla. The relative abundance of aphids in the genus *Aphis* and *R. padi* varied by year at Grimstad and Grue but were always amongst the four most numerous. *Rhopalosiphum padi* was most abundant in Stange, Stjørdal and Overhalla each year. A massive immigration of *R. padi* in Stange in August 2017 accounted for 87% of that year’s aphid count**.** The weekly species diversity is presented in Table S4.

The GLMM revealed that year and location were significant predictors of aphid abundance. Aphid counts were significantly higher in 2017 compared to 2016 (β = 0.553, SE = 0.233, p = 0.018), while counts in 2018 did not differ significantly from 2016 (β = 0.249, SE = 0.283, p = 0.379). Among locations, Grimstad served as the reference with the highest baseline counts. Overhalla (β =  − 1.589, SE = 0.367, p < 0.001), Stjørdal (β =  − 1.118, SE = 0.347, p = 0.001), and Grue (β =  − 0.833, SE = 0.299, p = 0.005) showed significantly lower counts, whereas Stange exhibited a non‑significant trend toward lower abundance (β =  − 0.631, SE = 0.375, p = 0.093). In contrast, temperature (β =  − 0.031, SE = 0.043, p = 0.471) and precipitation (β =  − 0.026, SE = 0.042, p = 0.537) did not show statistically significant effects on aphid abundance (Table S5).

### Occurrence of potential PVY and PVA vectors

Of the 111 aphid species identified with ≥ 98% blastn identity, 39 species are known vectors of PVY^22–24,28,29,42–48^, while nine species (8.1%) can transmit both PVY and PVA^24,49^. Aphid species capable of transmitting PVY and PVA were found in all sites and years with varying species composition, including the species recognized as key virus vectors (Table 2). The highest diversity of vector species occurred in the southernmost potato field.

**Table 2:** Aphid species recognized to be amongst the most important vectors of PVY and PVA, and that were recorded in the potato fields at the different locations in Norway in the period 2016—2018.

Vector species with potential transmitted virus	Collection site and year												
	Grimstad			Grue			Stange		Stjørdal			Overhalla	
	2016	2017	2018	2016	2017	2018	2016	2017	2016	2017	2018	2016	2017
*Acyrthosiphon pisum*^PVY^	x	x	x	–	x	x	x	x	–	–	–	x	x
Aphis fabae* ^PVY/PVA^	x	x	x	x	x	x	x	x	x	x	x	–	x
Aphis frangulae* ^PVY/PVA^	x	x	–	–	x	–	–	–	–	–	x	–	–
Aphis gossypii* ^PVY^	x	x	x	x	x	x	–	x	x	x	–	–	x
*Aphis spp.* ^PVY/PVA^	x	x	x	x	x	x	x	x	x	x	x	x	x
Aulacorthum solani* ^PVY/PVA^	–	x	–	x	–	–	–	–	–	x	–	–	x
*Brachycaudus helichrysi*^PVY^	x	x	–	–	–	–	–	–	–	–	–	–	–
*Capitophorus eleagni*^PVY^	x	–	–	–	–	–	–	–	–	–	–	–	–
*Cavariella aegopodii*^PVY/PVA^	x	x	–	x	x	–	–	–	–	–	–	–	x
*Hyperomyzus lactucae*^PVY^	x	x	x	–	x	–	–	x	–	x	x	–	–
Macrosiphum euphorbiae* ^PVY/PVA^	x	x	x	–	–	–	–	–	–	–	–	–	–
*Metopolophium dirhodum*^PVY/PVA^	x	–	–	–	x	–	x	x	–	x	x	–	–
Myzus ascalonicus* ^PVY^	–	x	–	–	–	–	–	–	–	–	–	–	–
Myzus persicae* ^PVY/PVA^	–	x	x	–	x	x	x	x	–	–	x	–	–
*Phorodon humuli*^PVY^	x	x	–	–	–	–	–	–	–	–	x	–	–
*Rhopalosiphum padi*^PVY/PVA^	x	x	x	x	x	x	x	x	x	x	x	x	x
*Sitobion avenae*^PVY/PVA^	x	x	x	–	x	x	x	x	–	–	–	–	x

Incomplete data for Stange 2016 and Stjørdal 2017.

x = recorded,–= not recorded.

*Colonizing species.

**Fig. 4:**
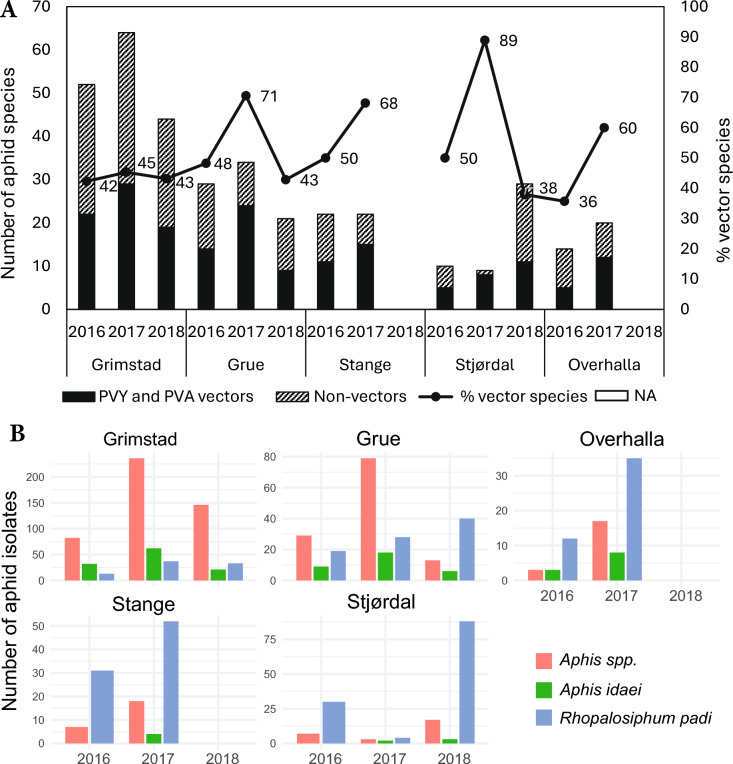
**P**otential aphid virus vectors in potato fields in Norway at locations Grimstad, Grue, Stange, Stjørdal and Overhalla in the growing seasons 2016–2018, during the period from the emergence of the potato seedlings to the haulm destruction. **A** Proportion of vector and non-vector aphid species. **B** The most interesting group and species that are known to be virus vectors. Aphids collected in the period 11/8 to 8/9–2016 in Stange and 19/6 to 14/8–2017 in Stjørdal were not identified to species. *NA* undetermined.

In total, 4283 of the collected specimens (70%) are considered potential PVY and PVA-vectors. Aphids of the genus *Aphis* were treated as a group in this estimate of vector abundance, since at least 19 *Aphis* species are recognised PVY and/or PVA vectors. Aphid abundance and species richness varied with season, site and year, as did the proportion of potential PVY and PVA vectors. Grimstad enclosed the lowest proportion of vectors each year (29% in 2016 and 58% in 2017 and 2018), while vector counts was generally similar or higher at the other sites. The proportion of potential vector species of the total number of aphid species was between 36 and 89% in the different sites and years (Fig. 4A).

Of the detected species recognized as important vectors (Table 2), the *Aphis-*group and *R. padi* was amongst the most abundant in all sites and years (Fig. 4B). The group of *Aphis*-species, even when the possibly non-vector *A. idaei* was excluded from the counting, was far more numerous than *R. padi* in Grimstad, whereas *R. padi* was the most abundant species in Stange, Stjørdal and Overhalla. In addition to the *Aphis*-group and *R. padi*, 4 to 12 other species regarded as important vectors was found at the different locations. *Myzus persicae* was recorded at all sites except Overhalla, but only 1–10 individuals were trapped per season. Other vectors were typically few in numbers, except occasionally higher catches of *Hyperomyzus lactucae* and *H. pallidus* (up to 95 specimens), *Macrosiphum euphorbiae* (up to 38 specimens), and *Metopolophium dirhodum* (up to 76 specimens) in some years and sites.

## Discussion

DNA barcoding targeting a ~ 650 bp fragment of the *COI* gene have proven essential to insect identification, and allowed us to identify aphid species from potato fields in Norway over a 3-years period. In this study, we found 137 different species, 111 species at a blastn threshold of ≥ 98% identity. The inability to identify 26 species (in 21 different genera) within the 2% species delimitation may therefore indicate unknown species within the genera. The identified aphids, were from nine sub-families and 54 genera, illustrating the huge diversity within this group. The strong bias towards A + T content in sequence data is also noteworthy, as it may have implications for how we interpret phylogenetic relationships. Furthermore, the range of intraspecific genetic distances raises questions about the potential for cryptic speciation or environmental influences on genetic variation. The variation observed in interspecific genetic distances highlights the need for caution in classifying genetically distinct aphid species. Overall, these findings enhance our understanding of aphid taxonomy and underscores the importance of genetic analysis in biodiversity studies.

Different sampling methods (such as suction traps and water pan traps) are used to monitor aphid flight in potato crops as part of virus risk assessment programs^22,49,50^. Suction traps effectively sample airborne aphids and provide early warning of vector activity around seed potato fields, but they do not necessarily reflect the aphids landing in the crop; in contrast, water pan traps placed at canopy level can better capture the aphids that actually land on plants^50^. In this study, yellow traps were chosen to avoid missing early or low-density aphid flights, but this choice may have overestimated the abundance and diversity of species strongly attracted to yellow while underrepresenting certain species that prefer green^50^.

This study shows that there is a large diversity of aphid species that migrate into potato fields during the potato growing season in Norway, and that the abundance, richness and species composition of potential vectors and non-vectors vary between sites and years. This agrees with findings in a four-year monitoring of aphid flight in seed potato fields in Tyrnävä-Liminka (northern Finland), which is one of the European High Grade Seed Potato Production Zones recognised by the European Union. The Finnish study revealed 84 taxa based on morphological identification and 34 species identified by barcoding^51^. In our study, the ten most dominating species in order of abundance was *Rhopalosiphum padi, Aphis idaei, Aphis fabae, Aphis rumicis, Amphorophora rubi, Hayhurstia atriplicis, Cryptomyzus galeopsidis, Aphis gossypii, Hyperomyzus lactucae and Euceraphis betulae* (Fig. 3). These species represented about 60% of the total aphid catch, and *Aphis* genus constituted 25%. As in Finland^51^, *R. padi* was often most abundant at the end of the potato growing season in our study (Fig. 4B), and was the most abundant species in Stange, Stjørdal and Overhalla and amongst the most abundant in Grimstad and Grue. The statistical analysis demonstrated that aphid abundance varied significantly by year and location, with notably higher counts in 2017 and reduced levels in Overhalla, Stjørdal, and Grue compared to Grimstad. In contrast, temperature and precipitation did not exhibit significant effects, suggesting that spatial and temporal factors may play a more dominant role in shaping aphid population dynamics during early-season weeks.

Aphids capable of transmitting PVY and PVA were found on all sites in our study. Of the 111 aphid species identified in this study, 39 of them have been reported as vectors of PVY^22–24,28,29,42–48^, while 9 of them have been reported to transmit PVA in addition to PVY^24,49^. In Finland, Kirchner et al.^25^ found that 37% of alate aphids caught in potato fields belonged to nine species reported to transmit PVY. In our study, 42% of the total alate aphid catch belonged to the 15 species listed as important PVY vectors, of which 54% also are reported to transmit PVA (Table 2). More recent research reveals little consensus about the relative importance of different aphid species in PVY epidemiology^26^, and several studies suggest that the total catch of colonizing and non-colonizing alate aphids, particularly in the early part of the potato growing season, may be a better indicator of PVY epidemiology than the occurrence of the aphid species considered to be the most efficient vectors^20,22,24,52–54^. High abundance of less efficient vectors for PVY can make them more important in virus epidemiology because of their frequent interplant movement and probing for suitable hosts, and especially if the immigration occurs when potato plants are young and susceptible to virus infection^22,26^. In Sweden, it was indicated that *R. padi* can be an important vector of PVY^55^, and Mondale et al.^53^ and Fox et al.^24^ suggest that *R. padi* have a higher PVY transmission efficiency than previously recognised. *Myzus persicae*, which is recognized as the most efficient vector of PVY, was found in low frequency in our study, in accordance with results from North-Central USA^20,25,52,56,57^ and Switzerland^26^.

Cereal aphids and other non-colonizing aphids could be more important as PVY vectors than *M. persicae* due to their higher abundance^26^. However, Steinger et al.^26^ found no correlation between the *R. padi* cumulative counts in suction traps from mid-April to mid-June and PVY post-harvest incidence in potatoes in Switzerland. Kirchner et al.^25,51^ suggested that *R. padi* may be of minor importance in northern regions like Finland due to their distinct monomodal, late flight activity, which peaks late in the growing season when mature plant resistance makes potatoes less susceptible to virus infection. In the study of Ryden et al.^55^ in Sweden, *R. padi* showed an early flight pattern (first part of the growing season) that coincided with early PVY spread, and it was suggested that this species played an important role in PVY transmission. Our study also identified *Aphis gossypii*, typically a greenhouse pest in Norway, at all field sites. This species has also been recorded in yellow pan traps and suction traps in Finland by Kirchner et al.^51^. In Switzerland, early cumulative catches of *Brachycaudus helichrysi* in suction traps were far the strongest predictor of post-harvest virus diseases (mainly PVY) in PVY-susceptible potatoes varieties, followed by *Phorodon humuli* and *Aphis* spp. whereas there was no correlation between virus incidence and *M. persicae* in spite of their often high abundance^26^. Steinger et al. (2015) pointed out that the early flight of considerable numbers of *B. helichrysi* coincidenced with a period of high potato susceptibility to virus infection, whereas alate *M. persicae* start to become more abundant later in the season^26^.

## Conclusion

This study provides new knowledge on the aphid fauna in potato production districts in South Norway and reveals a significant diversity of aphid species migrating into potato fields throughout the growing season. The highest total abundance and species diversity were consistently found at the southernmost site each year, while the proportion of potential PVY and PVA vector numbers and vector species tended to increase towards the north. Although the aphid abundance and species composition of potential vectors and non-vectors varied across different locations and years, the consistently relatively high proportion of potential PVY and PVA vectors shows that risk of virus infection and spread is always present. The need for and timing of virus control measures in seed potatoes are, however, dependent on the temporal vector flight activity when the potato plants is young and most susceptible for virus infection. Our research has provided new insights into the occurrence and phenology of vector and non-vector aphid species in various potato-growing regions in Norway. The data presented here can be used for future studies on the relationship between aphid flight activity and virus incidence which can enhance monitoring, risk assessment and management of aphid-borne virus diseases in seed potato crops.

## Supplementary Information

Supplementary Information 1.

Supplementary Information 2.

Supplementary Information 3.

Supplementary Information 4.

Supplementary Information 5.

## Data Availability

All data generated or analysed during this study are included in this published article (and its Supplementary Information files), and are available in the NCBI repository, accession numbers PV437596–PV439812.
