# Supplementary material for: Aphid populations and virus vector potential in potato fields across seasons and regions in Norway
Source: Sci Rep. 2025 Oct 21;15:36675. doi: 10.1038/s41598-025-20355-5 (PMC12540715; doi:10.1038/s41598-025-20355-5)
Supplement: Supplementary file 3 — Supplementary Information 3. [file 41598_2025_20355_MOESM3_ESM.docx]

**Table S3.** Aphid species collected in yellow water pan traps placed in potato fields at different locations and years.

| **Species** | **Collection site and year** | | | | | | | | | | | | |
| --- | --- | --- | --- | --- | --- | --- | --- | --- | --- | --- | --- | --- | --- |
|  | Grimstad | | | Grue | | | Stange | | Stjørdal | | | Overhalla | |
|  | 2016 | 2017 | 2018 | 2016 | 2017 | 2018 | 2016 | 2017 | 2016 | 2017 | 2018 | 2016 | 2017 |
| *Acyrthosiphon churchillense* Robinson 1979 |  | 5 | 1 |  |  | 1 | 1 |  |  |  |  |  |  |
| *Acyrthosiphon malvae* Mosley 1841 | 1 | 3 |  |  |  |  |  | 1 |  |  |  |  |  |
| *Acyrthosiphon pisum* Harris 1776 | 1 | 1 | 2 |  | 3 | 2 | 2 | 2 |  |  |  | 1 | 1 |
| *Amphorophora rubi* Kaltenbach 1843 | 21 | 31 | 41 | 2 | 3 |  |  | 1 |  |  |  | 1 | 10 |
| *Anoecia corni* Fabricius 1775 | 2 | 11 |  | 2 | 1 |  |  | 1 |  |  |  |  |  |
| *Anuraphis pyrilaseri* Shaposhnikov 1950 | 11 | 2 |  |  |  |  |  |  |  |  |  |  |  |
| *Aphis confusa* Walker 1849 | 3 | 4 |  |  |  |  |  |  |  |  |  |  |  |
| *Aphis coronillae* Ferrari 1872 |  | 1 | 1 | 1 | 3 | 2 | 1 | 1 |  |  | 2 |  | 1 |
| *Aphis craccae* Linnaeus 1758 |  |  | 5 |  | 1 |  |  |  |  |  | 1 |  |  |
| *Aphis craccivora* Koch 1854 | 4 | 1 | 4 |  | 7 | 1 |  |  | 1 |  |  |  | 1 |
| *Aphis cytisorum* Hartig 1841 | 1 | 2 | 1 |  |  |  |  |  |  |  |  |  |  |
| *Aphis fabae* Scopoli 1763 | 19 | 43 | 10 | 2 | 27 | 1 | 5 | 7 | 2 | 1 | 6 |  | 2 |
| *Aphis farinosa* Gmelin 1790 | 1 | 4 |  |  | 2 |  |  |  |  |  |  |  |  |
| *Aphis frangulae* Kaltenbach 1845 | 1 | 23 |  |  | 3 |  |  |  |  |  | 1 |  |  |
| *Aphis gossypii* Glover 1877 | 5 | 14 | 23 | 13 | 7 | 3 |  | 1 | 2 | 1 |  |  | 3 |
| *Aphis grossulariae* Kaltenbach 1843 |  |  | 1 |  |  |  |  |  |  |  |  |  |  |
| *Aphis idaei* van der Goot 1912 | 32 | 62 | 21 | 9 | 18 | 6 |  | 4 |  |  | 2 | 3 | 8 |
| *Aphis mamonthovae* Davletshina 1964 | 1 | 20 | 1 |  | 2 |  |  |  |  |  | 1 |  |  |
| *Aphis pomi* Vallot 1802 |  |  |  | 1 |  |  |  |  |  |  |  |  |  |
| *Aphis punicae* Passerini 1863 | 1 |  | 1 |  |  |  |  |  |  |  |  |  |  |
| *Aphis ruborum* Börner 1931 | 5 | 5 | 9 |  |  |  |  |  |  |  |  |  |  |
| *Aphis rumicis* Linnaeus 1758 | 4 | 30 | 66 | 1 | 2 |  |  | 4 |  | 1 | 4 |  | 2 |
| *Aphis sambuci* Linnaeus 1758 | 4 | 12 | 1 | 1 | 7 |  | 1 | 1 |  |  |  |  |  |
| *Aphis serpylli* Koch 1854 |  | 2 |  |  |  |  |  |  |  |  |  |  |  |
| *Aphis solanella* Theobald 1914 |  |  |  |  |  |  |  |  | 2 |  |  |  |  |
| *Aphis taraxacicola* Börner 1940 |  | 1 |  |  |  |  |  |  |  |  |  |  |  |
| *Aphis ulmariae* Schrank 1801 |  |  | 2 |  |  |  |  |  |  |  |  |  |  |
| *Aphis urticata* Gmelin 1790 | 1 | 12 |  | 1 |  |  |  |  |  |  |  |  |  |
| *Aulacorthum solani* Kaltenbach 1843 |  | 3 |  | 1 |  |  |  |  |  | 1 |  |  | 3 |
| *Brachycaudus cardui*  Linnaeus 1758 |  | 1 |  |  |  |  |  |  |  |  |  |  |  |
| *Brachycaudus helichrysi* Kaltenbach 1843 | 3 | 6 |  |  |  |  |  |  |  |  |  |  |  |
| *Brachycaudus rumexicolens* Patch 1917 | 1 |  |  |  |  |  |  |  |  |  |  |  |  |
| *Brachycaudus spiraeae* Börner 1932 |  |  |  | 2 |  |  |  |  |  |  |  |  |  |
| *Calaphis betulicola* Kaltenbach 1843 | 1 |  |  | 1 |  |  |  |  |  |  | 1 |  |  |
| *Calaphis flava* Mordvilko 1928 |  | 1 |  | 4 |  |  | 1 |  |  |  | 2 | 3 |  |
| *Callipterinella calliptera* Hartig 1841 |  |  |  |  |  | 1 |  |  |  |  |  |  |  |
| *Callipterinella tuberculata* von Heyden 1837 |  | 1 |  |  |  |  |  |  |  |  |  |  |  |
| *Capitophorus elaeagni* Del Guercio 1894 | 2 |  |  |  |  |  |  |  |  |  |  |  |  |
| *Capitophorus hippophaes* Walker 1852 |  | 1 | 1 |  |  |  |  |  |  |  |  |  |  |
| *Cavariella aegopodii* Scopoli 1763 | 3 | 3 |  | 6 | 3 |  |  |  |  |  |  |  | 1 |
| *Cavariella pastinacae* Linnaeus 1758 | 3 |  |  | 8 |  | 1 | 3 |  |  |  | 1 | 6 |  |
| *Cavariella theobaldi* Gillette & Bragg 1918 |  | 2 |  | 2 | 1 |  |  |  |  |  |  |  |  |
| *Chaitophorus nigricantis* Pintera 1987 |  |  |  |  |  |  |  |  |  |  | 2 |  |  |
| *Chaitophorus populialbae* Boyer de Fonscolombe 1841 |  | 2 | 7 |  |  | 1 |  |  |  |  |  |  |  |
| *Chaitophorus saliapterus* Shinji 1924 |  |  |  |  |  |  |  |  |  |  | 2 |  |  |
| *Chaitophorus tremulae* Koch 1854 |  |  |  |  |  |  | 1 |  |  |  |  |  |  |
| *Cinara pini* Linnaeus 1758 |  |  | 1 |  |  |  |  |  |  |  |  |  |  |
| *Cinara pilicornis* Hartig 1841 | 1 |  |  |  |  |  |  |  |  |  |  |  |  |
| *Clethrobius comes* Walker 1848 |  |  |  |  |  |  |  |  |  |  | 2 |  |  |
| *Coloradoa rufomaculata* Wilson 1908 |  |  |  |  |  |  |  |  |  |  | 1 |  |  |
| *Cryptomyzus galeopsidis* Kaltenbach 1843 | 3 | 26 | 3 | 2 | 8 |  | 2 | 11 | 4 |  | 6 | 8 | 5 |
| *Cryptomyzus ribis* Linnaeus 1758 |  | 20 |  |  | 1 |  | 1 |  |  |  |  |  |  |
| *Drepanosiphum platanoidis* Schrank 1801 |  |  |  |  |  |  |  |  |  |  | 1 |  |  |
| *Dysaphis angelicae* Koch 1854 |  |  | 1 |  |  |  |  |  |  |  |  |  |  |
| *Dysaphis plantaginea* Passerini 1860 |  | 1 |  |  | 1 |  |  |  |  |  |  |  |  |
| *Elatobium abietinum* Walker 1849 | 3 |  |  |  |  |  |  |  |  |  |  |  |  |
| *Ericaphis fimbriata* Richards 1959 | 1 |  |  |  |  |  |  |  |  |  |  |  |  |
| *Eriosoma anncharlotteae* Danielsson 1979 |  | 1 |  | 1 |  |  |  | 2 |  |  |  | 1 | 1 |
| *Eucallipterus tiliae* Linnaeus 1758 | 1 |  |  |  |  |  | 2 |  | 1 |  |  |  |  |
| *Euceraphis betulae* Koch 1855 | 1 | 21 |  | 19 |  |  | 7 | 2 | 1 |  |  | 4 |  |
| *Euceraphis papyrifericola* Blackman 2002 | 1 |  |  | 2 |  |  | 6 |  |  |  |  |  |  |
| *Euceraphis punctipennis* Zetterstedt 1828 |  | 1 |  | 5 |  |  | 8 |  |  |  |  |  | 3 |
| *Forda marginata* Koch 1857 |  | 1 |  |  |  |  |  |  |  |  |  |  |  |
| *Hayhurstia atriplicis* Linnaeus 1761 |  | 4 | 25 | 2 | 11 | 33 | 6 | 3 |  |  | 7 | 4 | 1 |
| *Hyalopterus pruni* Geoffroy *1762* |  |  | 6 |  | 1 |  |  |  |  |  |  |  |  |
| *Hyperomyzus lactucae* Linnaeus 1758 | 10 | 12 | 2 |  | 27 |  |  | 2 |  | 3 | 13 |  |  |
| *Hyperomyzus pallidus* Hille Ris Lambers 1935 |  | 2 |  | 1 | 7 |  |  | 1 |  |  |  |  |  |
| *Hyperomyzus rhinanthi* Schouteden1903 |  |  |  |  |  |  |  |  |  |  | 1 |  |  |
| *Jacksonia papillata* Theobald 1923 |  | 1 |  |  |  |  |  |  |  |  |  |  |  |
| *Lipaphis erysimi* Kaltenbach 1843 |  |  | 2 |  |  |  |  |  |  |  |  |  |  |
| *Macrosiphoniella artemisiae* Boyer de Fonscolombe 1841 | 2 | 2 |  |  | 1 |  |  |  |  |  |  |  |  |
| *Macrosiphoniella millefolii* De Geer 1773 |  |  |  | 1 |  |  | 1 |  |  |  |  |  | 1 |
| *Macrosiphoniella oblonga* Mordvilko 1901 | 3 | 1 | 3 |  |  |  |  |  |  |  |  |  |  |
| *Macrosiphoniella subterranea* Koch 1855 |  | 1 |  |  |  |  |  |  |  |  |  |  |  |
| *Macrosiphum euphorbiae* Thomas 1878 | 7 | 22 | 1 |  |  |  |  |  |  |  |  |  |  |
| *Macrosiphum funestum* Macchiati 1885 | 1 | 1 | 2 |  |  |  |  |  |  |  |  |  |  |
| *Macrosiphum rosae* Linnaeus 1758 |  |  |  |  |  |  |  |  |  |  | 1 |  |  |
| *Megoura viciae* Buckton 1876 |  |  | 2 |  |  | 1 | 2 |  |  |  |  |  |  |
| *Metopeurum fuscoviride* Stroyan 1950 |  |  |  | 1 |  |  |  |  |  |  |  |  |  |
| *Metopolophium dirhodum* Walker 1849 | 1 |  |  |  | 28 |  | 1 | 2 |  | 1 | 7 |  |  |
| *Microlophium carnosum* Buckton 1876 | 1 |  | 1 |  | 3 |  |  |  |  |  |  |  |  |
| *Muscaphis escherichi* Börner 1939 |  |  |  |  |  |  |  |  |  |  |  |  | 1 |
| *Myzocallis coryli* Goeze 1778 | 5 | 1 |  |  |  |  |  |  |  |  |  |  |  |
| *Myzus ascalonicus* Doncaster 1946 |  | 1 |  |  |  |  |  |  |  |  |  |  |  |
| *Myzus cerasi* Fabricius 1775 | 2 | 1 | 1 |  |  |  |  |  |  |  |  |  |  |
| *Myzus persicae* Sulzer 1776 |  | 6 | 1 |  | 3 | 3 | 1 | 1 |  |  | 1 |  |  |
| *Nasonovia ribisnigri* Mosley 1841 |  | 1 |  |  | 1 |  |  | 3 |  |  |  |  |  |
| *Pemphigus betae* Doane 1900 |  |  |  |  |  | 1 |  |  |  |  |  |  |  |
| *Pemphigus bursarius* Linnaeus 1758 |  | 9 | 3 |  | 5 | 2 |  | 1 |  |  |  |  |  |
| *Periphyllus testudinaceus* Fernie 1852 | 1 | 2 |  |  |  |  |  |  |  |  |  |  |  |
| *Phorodon humuli* Schrank 1801 | 1 | 2 |  |  |  |  |  |  |  |  | 1 |  |  |
| *Phyllaphis fagi* Linnaeus 1761 | 1 |  |  |  |  |  |  |  |  |  | 1 |  |  |
| *Pterocallis alni* De Geer 1773 | 3 | 3 | 7 | 1 |  |  |  |  |  |  | 23 | 1 | 1 |
| *Rhopalosiphum insertum* Walker 1849 | 1 |  |  |  | 2 |  |  |  |  | 1 |  |  |  |
| *Rhopalosiphum maidis* Fitch 1856 | 1 |  | 1 |  |  | 1 |  |  |  |  |  |  |  |
| *Rhopalosiphum padi* Linnaeus 1758 | 13 | 37 | 33 | 19 | 28 | 40 | 31 | 52 | 30 | 4 | 88 | 12 | 35 |
| *Schizaphis graminum* Rondani 1852 |  |  |  |  |  |  |  |  |  |  |  | 2 | 1 |
| *Sitobion avenae* Fabricius 1775 | 6 | 4 | 4 |  | 3 | 1 | 3 | 2 |  |  |  |  | 1 |
| *Tetraneura ulmi* Linnaeus 1758 | 1 | 1 |  |  |  |  |  |  |  |  |  |  |  |
| *Thecabius affinis* Kaltenbach 1843 |  |  | 5 |  |  | 1 |  |  | 6 |  | 1 | 6 |  |
| *Thelaxes dryophila* Schrank 1801 |  | 1 | 2 |  |  |  |  |  |  |  |  |  |  |
| *Therioaphis trifolii* Monell 1882 |  |  | 2 |  |  | 3 |  |  |  |  |  |  |  |
| *Toxopterina vandergooti* Börner 1933 |  |  |  |  |  | 1 |  |  |  |  |  |  |  |
| *Tuberculatus annulatus* Hartig 1841 | 4 | 10 |  |  |  |  |  |  |  |  |  |  |  |
| *Tuberculatus querceus* Kaltenbach 1843 |  | 1 | 1 |  |  |  |  |  |  |  |  |  |  |
| *Tuberculatus remaudierei* Nieto Nafría 1974 |  | 3 |  |  |  |  |  |  |  |  |  |  |  |
| *Uroleucon achilleae* Koch 1855 | 1 | 6 |  |  |  |  |  |  |  |  |  |  |  |
| *Uroleucon cirsii* Linnaeus 1758 |  |  | 1 |  |  |  |  |  | 1 |  |  |  |  |
| *Uroleucon hypochoeridis* Fabricius 1779 |  |  |  |  |  |  |  |  |  |  | 1 |  |  |
| *Uroleucon sonchi* Linnaeus 1767 |  |  |  |  | 1 |  |  |  |  |  |  |  |  |
| *Uroleucon taraxaci* Kaltenbach 1843 |  |  |  |  | 1 |  | 1 |  |  |  | 1 | 1 |  |
| **Number of species** | **52** | **64** | **43** | **29** | **34** | **21** | **22** | **22** | **10** | **8** | **29** | **14** | **20** |

**Table S3.** Aphid species collected in yellow water pan traps placed in potato fields at different locations and years. The values are number of specimens barcoded. Identification is based on a species limitation threshold of 2.0 %. Incomplete data for Stange 2016 and Stjørdal 2017 (parts of the growing season not covered).
