# Supplementary material for: Aphid populations and virus vector potential in potato fields across seasons and regions in Norway
Source: Sci Rep. 2025 Oct 21;15:36675. doi: 10.1038/s41598-025-20355-5 (PMC12540715; doi:10.1038/s41598-025-20355-5)
Supplement: Supplementary file 5 — Supplementary Information 5. [file 41598_2025_20355_MOESM5_ESM.docx]

**Table S5.** Model coefficients (log scale), random effects, and fit statistics.

Negative binomial (NB2) GLMM with log link: Aphids ~ Temperature + Percipitation + factor(Year) + Location + (1 | Location:Year). Coefficients (β) are on the log scale as reported by glmmTMB. Reference categories: Year 2016 and Location Grimstad. N = 152 observations; 13 Location:Year groups.

Fixed effects

| Predictor | Beta | SE | z | p |
| --- | --- | --- | --- | --- |
| (Intercept) | 4.60533 | 0.85055 | 5.414 | 6.15e-08 |
| Temperature | -0.03132 | 0.04349 | -0.720 | 0.47147 |
| Percipitation | -0.02609 | 0.04223 | -0.618 | 0.53668 |
| Year 2017 (vs 2016) | 0.55275 | 0.23291 | 2.373 | 0.01763 |
| Year 2018 (vs 2016) | 0.24931 | 0.28332 | 0.880 | 0.37888 |
| Grue (vs Grimstad) | -0.83298 | 0.29930 | -2.783 | 0.00538 |
| Overhalla (vs Grimstad) | -1.58871 | 0.36714 | -4.327 | 1.51e-05 |
| Stange (vs Grimstad) | -0.63118 | 0.37544 | -1.681 | 0.09273 |
| Stjørdal (vs Grimstad) | -1.11810 | 0.34682 | -3.224 | 0.00126 |

Random effects (Location:Year)

| Component | Variance | SD |
| --- | --- | --- |
| Intercept (Location:Year) | 0.02125 | 0.1458 |

Model fit and dispersion

| Metric | Value |
| --- | --- |
| AIC | 1405.6 |
| BIC | 1438.9 |
| logLik | -691.8 |
| NB2 dispersion parameter | 0.861 |
| Observations (N) | 152.0 |
| Groups (Location:Year) | 13.0 |
